# *Akkermansia muciniphila* and environmental enrichment reverse cognitive impairment associated with high-fat high-cholesterol consumption in rats

**DOI:** 10.1080/19490976.2021.1880240

**Published:** 2021-03-08

**Authors:** Sara G. Higarza, Silvia Arboleya, Jorge L. Arias, Miguel Gueimonde, Natalia Arias

**Affiliations:** a Laboratory of Neuroscience, Department of Psychology. University of Oviedo, Oviedo, Asturias, Spain; b Instituto De Neurociencias Del Principado De Asturias (INEUROPA), Asturias, Spain; c Department of Microbiology and Biochemistry of Dairy Products, Instituto De Productos Lácteos De Asturias (IPLA-CSIC), Villaviciosa, Asturias, Spain; d UK Dementia Research Institute, Department of Basic and Clinical Neuroscience, Maurice Wohl Clinical Neuroscience Institute, Institute of Psychiatry, Psychology and Neuroscience, King’s College London, London, UK

**Keywords:** NASH, cognition, novel object recognition, working memory, gut microbiota, microbiota–gut–brain axis, environmental enrichment, probiotics

## Abstract

Nonalcoholic steatohepatitis (NASH) is one of the most prevalent diseases globally. A high-fat, high-cholesterol (HFHC) diet leads to an early NASH model. It has been suggested that gut microbiota mediates the effects of diet through the microbiota–gut–brain axis, modifying the host’s brain metabolism and disrupting cognition. Here, we target NASH-induced cognitive damage by testing the impact of environmental enrichment (EE) and the administration of either *Lacticaseibacillus rhamnosus* GG (LGG) or *Akkermansia muciniphila* CIP107961 (AKK). EE and AKK, but not LGG, reverse the HFHC-induced cognitive dysfunction, including impaired spatial working memory and novel object recognition; however, whereas AKK restores brain metabolism, EE results in an overall decrease. Moreover, AKK and LGG did not induce major rearrangements in the intestinal microbiota, with only slight changes in bacterial composition and diversity, whereas EE led to an increase in Firmicutes and Verrucomicrobia members. Our findings illustrate the interplay between gut microbiota, the host’s brain energy metabolism, and cognition. In addition, the findings suggest intervention strategies, such as the administration of AKK, for the management of the cognitive dysfunction related to NASH.

## Introduction

Nonalcoholic fatty liver disease (NAFLD) is characterized by hepatic fat accumulation, and is closely associated with central obesity, diabetes, and other features of metabolic syndrome.^1^ NAFLD encompasses a broad disease spectrum, ranging from simple steatosis to nonalcoholic steatohepatitis (NASH), which may, in turn, develop into cirrhosis, end-stage liver disease, or hepatocarcinoma.^2^ Although NASH has become a serious global health threat, therapeutic strategies that could prevent NAFLD-NASH progression have been overlooked.

We previously published^3^ a study showing that consumption of a high-fat, high-cholesterol diet leads to an early NASH model that replicates the typical features of the human disease, including hepato- and splenomegaly, early NASH histopathology, hypercholesterolemia, increased serum liver enzymes, and increased pro-inflammatory cytokines.^3,4^ Studies have reported that a high-fat, high-cholesterol diet and a high-fat diet produce changes in the gut microbial composition, in addition to reductions in microbial diversity and changes in specific bacterial taxa.^3,5^ A recent study by Hoyles et al.^6^ showed that fecal transplantation from human donors with hepatic steatosis triggered rapid development of hepatic steatosis in mice, which highlights the contributing role of microbiota in NASH development.

Gut microbiota have also been found to play an important role in the central nervous system (CNS) through the microbiota–gut–brain axis, and CNS dysfunction has been linked to several psychiatric and non-psychiatric disorders.^7^ Although some studies have shown that short-chain fatty acids (SCFAs) can not only stimulate vagus nerve signaling, but also alter levels of neurochemicals such as serotonin,^8–10^ in a previous study we demonstrated that gut dysbiosis and decreased production of SCFAs found in NASH animals, acting through the gut–brain axis, were associated with a prefrontal dopamine depletion only, and not serotonin.^3^ We have also found that the dopaminergic dysfunction could be due to the reduced energy in the NASH group, which is dependent on an altered glucose metabolism, and also a consequence of the insulin resistance found in these animals.^3^

Moreover, neuronal communication requires a high amount of energy, which could be measured by labeling cytochrome c oxidase (CCO), a mitochondrial enzyme involved in ATP production and a reliable marker of brain energy demands.^11,12^ Thus, it can be used to detect regional brain differences in the metabolic capacity in response to cognitive processes.^13,14^

Furthermore, it has been demonstrated that CCO activity reflects the neuronal functional activity occurring over long time periods ranging from hours to weeks. Hence, this technique evaluates sustained or long-term changes in brain regional oxidative metabolic capacity.^11^ Indeed, several studies, from our group and others, have used CCO histochemistry to prove changes in CCO activity and CCO connectivity in several rat brain regions.^15–17^

These findings highlight the functional connection between the liver–gut microbiota and cognition. Thus, gut microbiota could be a target for improving cognitive deficiencies associated with NASH, with probiotics a promising tool for this purpose. However, the impact of probiotic administration on cognitive function is not well understood.

In addition, an inappropriate diet and physical inactivity often co-exist in these patients. Exercise is able to modulate the gut microbiota. Clarke et al.^18^ described that athletes showed a higher diversity of gut microorganisms, an observation also supported by other studies.^19,20^ In the brain, studies in elderly people have shown that exercise increases hippocampal volume and enhances hippocampus-dependent learning and memory.^21^ The effect of physical exercise in rodents is modeled using environmental enrichment (EE), which induces a situation of increased motor stimulation and sensorial and cognitive enhancement.^22^ EE has been shown to be associated with synaptic function and cellular plasticity changes, resulting in cognitive enhancement.^22–24^ Therefore, EE could mitigate the negative impact of a high-fat, high-cholesterol diet on the brain and cognition.

In the present study, we target high-fat, high-cholesterol (HFHC)-induced cognitive disturbances through the microbiota–gut–brain axis using two approaches: first, we assess the effects of EE as a cognitive enhancer, and second, we evaluate the impact of two specifically selected strains of probiotics, *Lacticaseibacillus rhamnosus* GG (LGG) and *Akkermansia muciniphila* CIP107961 (AKK), on NASH pathology. These strains have been selected based on previous studies where we observed that the gut microbiota in NASH rats suffered an important loss of *Lactobacillus* compared to the normal chow (NC) group. Because of this, we decided to test a *Lactobacillus* strain as a potential probiotic, and we selected the widely used probiotic strain *L. rhamnosus* GG,^25^ which has previously been demonstrated to positively modulate liver fatty acid composition in mice receiving a high-fat diet.^26^ Moreover, in a previous pilot study, we also tested the effect of EE on the gut microbiota and cognitive aberrances of a pilot NASH rat model, and observed that EE increased the levels of *Akkermansia* in the HFHC+EE group and ameliorated the NASH symptoms, compared to the HFHC control group. Therefore, we also decided to include the potentially novel probiotic *Akkermansia muciniphila*, which has been found in different animal models to ameliorate metabolic syndrome features such as obesity, diabetes, and cardiovascular diseases,^27^ enhance the lifespan in a progeria mice model,^28^ and improve amyotrophic lateral sclerosis symptoms.^29^ Moreover, in a recent human intervention study, *A. muciniphila* was found to improve liver function and metabolic markers.^30^ In both cases, we compare their effects on microbiota changes, brain metabolism, and, ultimately, cognitive improvement.

## Results

### Environmental enrichment restores cognitive deficits caused by the HFHC diet

**Figure 1.**
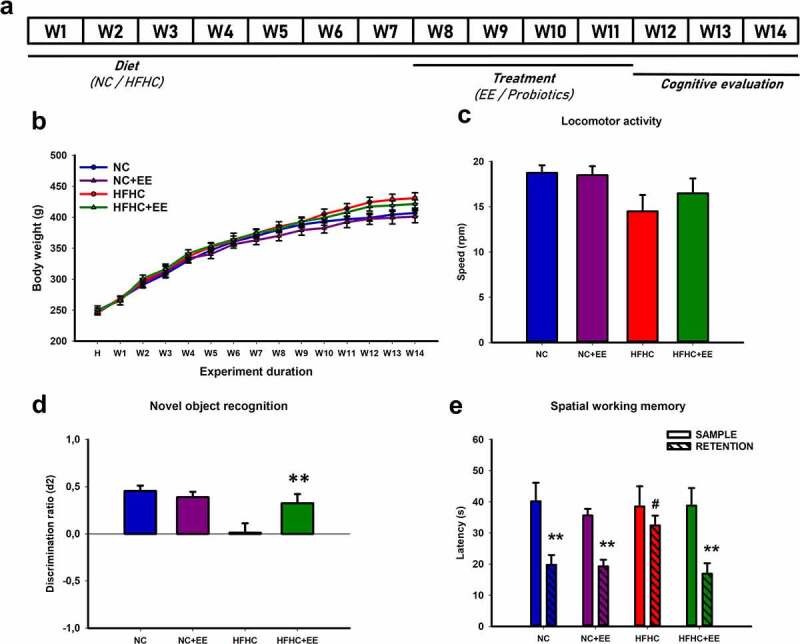
Timeline, body weight, and behavioral assessment of the experimental groups subjected to diet and EE. (a) Timeline of the experimental design. Each group was fed their respective diet for 14 weeks. From week 8 to 12, NC+EE and HFHC+EE were subjected to EE, whereas HFHC+LGG and HFHC+AKK were given their respective probiotic daily, and HFHC+PBS received phosphate buffered saline. From weeks 12 to 14, the cognitive evaluation took place, at the end of which the animals were sacrificed and the samples were collected. (b) **Body weight across weeks**. Two-way ANOVA (Group × Week) was used to assess weight gain. No changes in body weight between groups were found throughout the 14 weeks of the administration of the diet, and significant differences across the weeks were revealed. (c) **Locomotor function evaluation measured on the Rotarod-accelerod test**. Bar charts (mean ± SEM) represent the maximum speed (rpm) of the animals on the rod, compared with the Kruskal–Wallis test. There were no statistically significant differences between groups. (d) **Novel object recognition test**. Bar charts (mean ± SEM) represent the discrimination ratio (D2) between the new object and the one previously observed. Two-way ANOVA followed by Tukey’s test (#*p*< .05, **p≤ 0.010, ***p< .001) comparison of NC, NC+EE, HFHC and HFHC+EE D2 value were used. NC and NC+EE groups were able to recognize the new object; whereas HFHC was not able to discriminate the object, HFHC+EE showed a recovered novel object recognition ability. (e) **Spatial working memory test**. Bar charts (mean ± SEM) represent the average latency on the sample and retention trials. Two-tailed paired *t*-tests (* comparison with its respective sample) and one-way ANOVA followed by Tukey’s test (# comparison between NC, NC+EE and HFHC+EE) were used. NC and NC+EE groups remembered the position of the platform in the retention trial; whereas HFHC was not able to remember it, HFHC+EE displayed recovered spatial working memory. #*p*< .05, **p≤ 0.010

No statistically significant differences were found in the body weight between groups throughout the 14 weeks of administration of the diet (Figure 1(a)) (*p*= .306). As expected, significant differences between weeks were revealed (*p*< .001), with normal weight gain according to age in both experimental groups (Figure 1(b)). The normal increase in weight was accompanied by the absence of statistically significant locomotor deficits in all groups (*p*= .151; Figure 1(c)).

When the experimental groups performed the novel object recognition task, the two-way ANOVA revealed no statistically significant differences in the exploratory preference time in the acquisition/sample (E1; diet: *F*_1,31_ = 0.0308, *p*= .583; treatment: *F*_1,31_ = 0.000699, *p*= .079; interaction: *F*_1,31_ = 0.308, *p*= .583), either in the retention/test phase (E2; diet: *F*_1,31_ = 3.834, *p*= .060; treatment: *F*_1,31_ = 1.860, *p*= .183; interaction: *F*_1,31_ = 0.114, *p*= .738) between diets (NC or HFHC) or treatments (without or with EE), which means that these animals spent a similar amount of time exploring the objects in each phase of the test. Moreover, no differences were found in the exploratory preference indexes between acquisition/sample and retention/test phases in the NC (*F*_1,15_ = 0.676; *p*= .425), HFHC (*F*_1,15_ = 0.00569; *p*= .941), NC+EE (*H*_1_ = 2.019; *p*= .161), and HFHC+EE (*F*_1,15_ = 4.617; *p*≤ 0.050), which indicates that each group spent a similar amount of time exploring in the first and second phases of the test (Supplemental Figure 1(a)).

However, when we compared the discrimination index (D1), which is the difference in the time spent exploring the two objects in the test phase, we found an effect caused by the diet (*F*_1,31_ = 15.321, *p*< .001) but not by the treatment (*F*_1,31_ = 0.838, *p*= .368) or due to the interactions between diet and treatment (*F*_1,31_ = 4.023, *p*= .055; Supplemental Figure 1(a)). These results indicate that the NC group was able to discriminate between a previously encountered object and a novel object, whereas the HFHC group was not able to distinguish the new object.

After the implementation of EE, we found differences in the discrimination ratio (D2), which makes reference to the difference in the exploration time of the objects divided by the total time spent in exploration of the objects, not only due to the diet (*F*_1,31_ = 9.838, *p*= .004), but also the interactions between diet and treatment (*F*_1,31_ = 5.529, *p*= .026), where the HFHC+EE group was now able to recognize the novel object (*p*< .011). Thus, the HFHC group initially displayed an object recognition impairment that was reversed by the introduction of EE (Figure 1(d)).

Then, the animals were tested on a spatial working memory task. Whereas the NC group was able to remember the position of the platform, showing a statistically significant lower latency in the retention trial compared to the sample trial (*p*= .003), the HFHC group did not show statistically significant differences between the sample and retention trials (*p*= .333). After EE, the NC+EE maintained their capacity to remember the position of the platform (*p*= .002), and HFHC+EE were now also able to remember it (*p*= .002). Our results show that the HFHC impaired spatial working memory was ameliorated by the EE. When comparing the mean latencies in each trial between groups, we observed that, although the sample latencies were statistically similar (*p*= .941), the retention latencies differed (*p*= .008) because they were significantly higher in HFHC than in NC, NC+EE, and HFHC+EE (Figure 1(e)).

### Environmental enrichment cognitive improvement is accompanied by a decrease in brain metabolic activity

The HFHC group showed significantly lower CCO activity values than the NC group, with less metabolic activity in the infralimbic cortex (IL; *p*< .001), cingulated cortex (Cg, *p*< .001), dorsal striatum (dST, *p*< .001), accumbens shell (AcbS; *p*< .001), and perirhinal cortex (PRh; *p*= .002; Table 1). EE led to a decline in the CCO levels in the NC+EE and HFHC+EE groups. The NC+EE group displayed lower CCO levels than the NC group in the prefrontal cortex (*p*< .001), dorsal and ventral striatum (*p*< .001), thalamus (*p*< .001), and CA3 (*p*= .001) and DG (*p*< .001) hippocampal subregions. In addition, HFHC+EE animals also presented decreased CCO values compared to HFHC animals in the dorsal and ventral striatum (*p*< .001), anterodorsal thalamus (ADT; *p*< .001), basolateral amygdala (BLA; *p*= .006), dentate gryus (DG; *p*< .001), and CA3 (*p*= .001). Finally, HFHC+EE showed lower CCO values than NC+EE in the DG (*p*< .001). No differences were found between HFHC+EE and NC+EE in the prefrontal cortex, dorsal and ventral striatum, thalamus, amygdala, and perirhinal cortex.

**Table 1.** Brain oxidative metabolism in different groups subjected to NC and HFHC diets and EE. The CCO values are expressed as mean ± SEM. The studied regions included the prefrontal cortex (prelimbic (PrL), infralimbic (IL) and cingulate (Cg) cortex), the dorsal striatum (dST), the ventral striatum (accumbens core (AcbC) and shell (AcbSh)), the thalamus (anteromedial nucleus (AMT), anterodorsal nucleus (ADT) and anteroventral nucleus (AVT)), the amygdala (central (CeA), basolateral (BLA) and lateral (LaA)), the dorsal hippocampus (dentate gyrus (DG), CA1 and CA3 areas), and the perirhinal (PRh) and entorhinal (Ent) cortices. Data were analyzed using one-way ANOVA followed by Tukey’s test (*#&*p*< .05; * NC+EE and HFHC vs. NC; # HFHC+EE vs. HFHC; and HFHC+EE vs. NC+EE)

Region	NC	NC+EE	HFHC	HFHC+EE
**PrL**	28.526 ± 1.327	*21.506 ± 1.501	21.815 ± 1.042	* 19.999 ± 0.862
**IL**	28.153 ± 1.109	* 21.433 ± 1.323	* 22.027 ± 0.939	* 19.634 ± 0.930
**Cg**	28.846 ± 1.333	* 22.008 ± 1.283	* 22.684 ± 1.164	* 20.495 ± 0.791
**dST**	28.040 ± 0.707	* 22.115 ± 0.622	* 23.078 ± 0.671	*# 19.889 ± 0.784
**AcbC**	35.734 ± 1.337	* 26.434 ± 1.192	31.252 ± 0.903	*# 22.246 ± 0.780
**AcbSh**	39.684 ± 1.055	* 29.814 ± 1.122	* 34.000 ± 1.150	*# 25.962 ± 0.693
**AMT**	26.087 ± 1.280	* 16.413 ± 2.136	22.203 ± 0.694	* 17.540 ± 0.824
**ADT**	37.683 ± 1.684	* 28.188 ± 1.054	33.995 ± 1.348	*# 25.138 ± 1.207
**AVT**	31.661 ± 1.702	* 22.988 ± 1.793	27.684 ± 1.193	* 22.094 ± 0.700
**CeA**	23.299 ± 0.984	19.247 ± 2.411	22.482 ± 1.040	* 16.578 ± 1.151
**BLA**	25.912 ± 1.338	23.639 ± 1.334	25.180 ± 0.988	*# 19.254 ± 1.343
**LaA**	20.135 ± 0.828	20.268 ± 2.077	20.049 ± 0.641	14.897 ± 1.085
**DG**	34.185 ± 1.729	* 27.210 ± 0.723	30.501 ± 0.954	*#& 22.593 ± 0.770
**CA1**	20.905 ± 1.154	16.481 ± 1.709	17.978 ± 0.805	* 14.541 ± 1.231
**CA3**	20.556 ± 1.127	* 15.018 ± 1.583	18.396 ± 0.973	*# 13.403 ± 1.072
**PRh**	25.015 ± 1.240	15.018 ± 1.583	* 18.891 ± 0.896	* 17.205 ± 1.147
**Ent**	21.212 ± 1.502	17.704 ± 1.836	17.312 ± 0.760	16.035 ± 1.391

### Environmental enrichment has an effect on microbiota composition and bacterial metabolism

**Figure 2.**
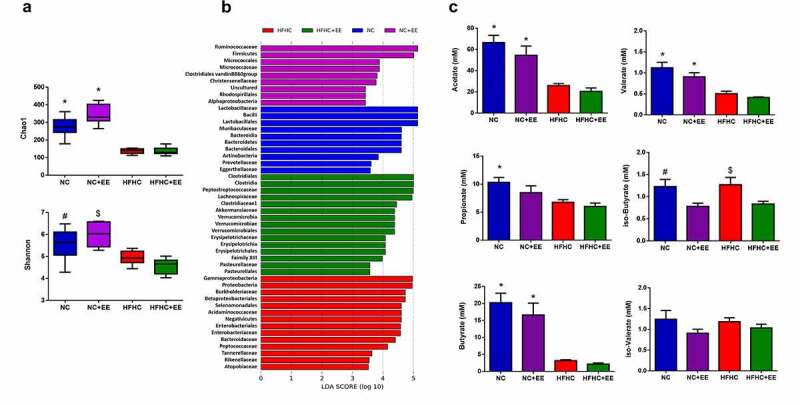
Gut microbiota exploration of different groups subjected to NC and HFHC diets and the implementation of environmental enrichment (EE). (a) Bacterial diversity. Box and whiskers (median and IRQ range) represent comparison of alpha-diversity of gut microbiota using Chao1 and Shannon indexes among groups studied, which was compared using a one-way ANOVA followed by Tukey test (* comparison with all the groups; # comparison with HFHC+EE group; $ comparison with HFHC and HFHC+EE groups). *#$*p*< .01. (b) **Gut microbiota composition**. Results of LEfSe analysis (LDA (Linear Discriminant Analysis) scores >2 and significance of *p*< .05 as determined by Wilcoxon’s signed-rank test) showing bacterial taxa with differentially abundance among the groups studied. Red indicates differential abundances in HFHC group; green indicates differential abundances in HFHC+EE group; blue indicates differential abundances in NC group; purple indicates differential abundances in NC+EE group. (c) **SCFA and BCFAs**. Bars charts (mean ± SEM) represent comparison of the SCFA and BCFA levels (mM) compared using the Kruskal–Wallis test followed by Dunn’s analysis (* comparison with HFHC and HFHC+EE groups; # comparison with NC+EE group; $ comparison with NC+EE and HFHC+EE groups). *#$*p*< .05

To assess the effect of the environmental enrichment (EE) implementation on the gut microbiota, we studied the gut microbiome profile of the NC+EE and HFHC+EE groups by comparing them with the profiles of the control groups (NC and HFHC) and with each other. We first studied how EE affects microbial diversity within the communities by calculating the Chao1 (richness estimator) and Shannon’s index (richness and evenness estimator). We confirmed significantly (*p < *.000) less bacterial diversity in the HFHC group compared to the NC group, as previously described in the characterization of the NASH animal model used,^3^ and we observed an increase in bacterial diversity in the NC+EE group compared to the NC group (*p < *.01). However, EE did not affect the bacterial diversity in the HFHC+EE group, which was significantly lower (also for evenness) than in the NC+EE group (*p < *.000) (Figure 2(a)).

Next, we assessed the gut microbiota composition in each group of rats. When comparing the NC and HFHC groups, the pattern was substantially different (Supplemental Figure 2), in agreement with previous studies.^3^ The main differences were found in the greater abundance of *Lactobacillaceae* and *Ruminococacceae* in the NC groups compared to the HFHC groups, which harbored a greater abundance of *Enterobacteriaceae, Bacteroidaceae*, or *Peptostreptococcaceae*. Then, to explore how EE could affect the different phylotypes of the microbiota, we applied a linear discriminant analysis effect size (LEfSe) method at the family level to investigate the taxa most likely to explain differences in abundances across the groups. When we compared the four animal groups – NC, NC+EE, HFHC, HFHC+EE – the results identified statistically significant increased abundance of *Micrococcaceae, Christensenellaceae*, and *Ruminococcaceae* in the NC+EE group compared to the remaining groups, and an increased abundance of different families belonging to the Firmicutes phyla and *Akkermansiaceae* as the most differential microorganisms in the HFHC+EE when compared with the other three groups (Figure 2(b)).

To explore the metabolic implications of the microbial differences observed after EE implementation, we analyzed the main SCFAs and branched-chain fatty acids (BCFAs) derived from the bacterial metabolism. First, when comparing the HFHC group and the NC group, we confirmed the previous observations,^3^ in which the main SCFAs (acetate, *p < *.01; propionate, *p < *.05; and butyrate, *p < *.01) showed statistically significant lower concentrations in the HFHC group. Then, the comparisons of NC+EE and HFHC+EE with their control groups (NC and HFHC, respectively) did not show any significant differences in acetate, propionate, butyrate, or valerate, even though a decreasing tendency was observed after EE. By comparison, the concentrations of the BCFAs iso-butyric (*p < *.05) and iso-valeric (n.s.) were lower in NC+EE and HFHC+EE, compared to NC and HFHC, respectively (Figure 2(c)).

### Akkermansia muciniphila, as a probiotic treatment, restores cognition deficits

**Figure 3.**
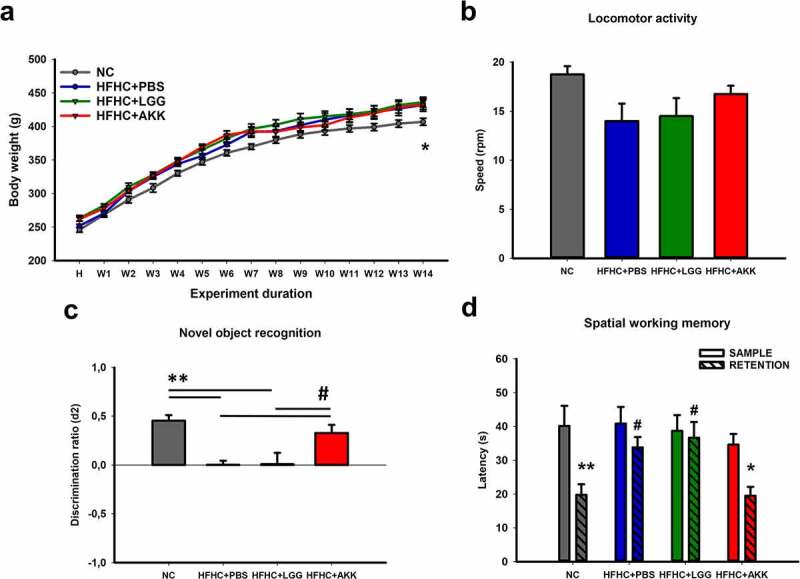
Body weight and behavioral assessment in experimental groups subjected to PBS, LGG, and AKK. (a) Body weight across experimental weeks. Two-way ANOVA (Group × Week) was used to assess weight gain. Slight significant changes in body weight between groups were found during the 14 weeks of the administration of the diet because the HFHC+LGG group gained more weight than the NC group; significant differences across the weeks were also revealed. *p< .05. (b) **Locomotor function evaluation measured on Rotarod-accelerod test**. Bar charts (mean ± SEM) represent the maximum speed (rpm) of the animals on the rod, compared using the Kruskal–Wallis test. There were no statistically significant differences between groups. (c) **Novel object recognition test**. Bar charts (mean ± SEM) represent the discrimination ratio (D2) between the new object and the one previously observed. Two-way ANOVA followed by Tukey’s test (#*p*< .05, **p≤ 0.010, ***p< .001) comparison of NC, HFHC+PBS, HFHC+LGG, and HFHC+AKK d2 value were used. The NC group was able to recognize the new object; whereas HFHC+PBS and HFHC+LGG were not able to discriminate it, HFHC+AKK showed a recovered novel object recognition ability. (d) **Spatial working memory test**. Bar charts (mean ± SEM) represent the average latency on sample and retention trials. Two-tailed paired *t*-tests (* comparison with its respective sample) and one-way ANOVA followed by Tukey’s test (# comparison between NC, HFHC+PBS, HFHC+LGG, and HFHC+AKK) were used. The NC group remembered the position of the platform on the retention trial; whereas HFHC+PBS and HFHC+LGG were not able to remember it, HFHC+AKK displayed recovered spatial working memory. *#*p*< .05, **p≤ 0.010

Slight changes were found in the body weights of the groups during the 14 weeks of the administration of the diet because the HFHC+LGG group gained more weight than the NC group (*p*= .038); significant differences were observed across weeks (*p*< .001), as expected (Figure 3(a)). The normal increase in weight was accompanied by the absence of locomotor deficits in all groups (*p*= .092; Figure 3(b)). In the object recognition test, the experimental groups did show significant differences in E1 (*F*_3,31_ = 5.871, *p*= .003) where HFHC+PBS (*p*= .002) and HFHC+LGG (*p*= .027) spent more time exploring than NC. Moreover, differences in E2 were found (*F*_3,31_ = 4.411, *p*= .012) where HFHC+LGG groups spent more time exploring than NC (*p*= .007) while performing the novel object recognition retention/test trial. Furthermore, no differences were found in the exploratory preference indexes between acquisition/sample and retention/test phases in the NC (*F*_1,15_ = 0.676; *p*= .425), HFHC (*F*_1,15_ = 0.386; *p*= .544), NC+EE (*F*_1,15_ = 0.190; *p*= .669), and HFHC+EE (*F*_1,15_ = 2.438; *p*= .141).

However, during the retention/test trial no differences were found between groups in D1 (*F*_3,31_ = 2.768, *p*= .060; Supplemental Figure 1(b)). When we compared the discrimination ratio between the groups, we found that NC and HFHC+AKK showed significantly higher D2 than HFHC+PBS (*p*= .003 vs NC; *p*= .038 vs HFHC+AKK) and HFHC+LGG (*p*= .003 vs NC; *p*= .043 vs HFHC+AKK). These results show that HFHC+PBS displayed an object recognition impairment that was not recovered by the administration of LGG, but was reversed by AKK (Figure 3(c)).

Finally, when we evaluated the efficacy of probiotic administration on a relevant prefrontal dependent task such as spatial working memory, we found that the HFHC+LGG group was unable to execute the task (*p*= .486) as well as the HFHC+PBS group (*p*= .103). However, the HFHC+AKK group performed the task (*p*= .019) as correctly as the NC group, and they were able to remember the position of the platform because they showed a statistically significant lower retention latency compared to the sample trials (*p*= .003). When we compared the mean latencies in each trial between groups, we observed that, although the sample latencies were statistically similar (*p*= .798), the retention latencies differed (*p*= .003) because they were significantly higher in HFHC+PBS and HFHC+LGG than in NC and HFHC+AKK (Figure 3(d)). These results highlight the efficacy of *A. muciniphila* CIP107961 as a probiotic treatment to reverse impaired spatial working memory, compared to other probiotics such as *L. rhamnosus* GG.

### Akkermansia muciniphila restores brain metabolic activity to normal

When we explored the brain metabolic activity underlying these cognitive changes, we first found that the HFHC+PBS group showed significantly lower levels of CCO activity than the NC group in the prefrontal cortex (*p*< .001), dorsal and ventral striatum (*p*< .001), and amygdala nuclei, such as CeA (*p*= .004), BLA (*p*= .014), hippocampus (*p*≤ 0.007), and PRh (*p*< .001; Table 2).

**Table 2.** Brain oxidative metabolism in experimental groups subjected to PBS, LGG and AKK. The CCO values are expressed as mean ± SEM. The studied regions included the prefrontal cortex (prelimbic (PrL), infralimbic (IL) and cingulate (Cg) cortex), the dorsal striatum (dST), the ventral striatum (accumbens core (AcbC) and shell (AcbSh)), the thalamus (anteromedial nucleus (AMT), anterodorsal nucleus (ADT) and anteroventral nucleus (AVT)), the amygdala (central (CeA), basolateral (BLA) and lateral (LaA)), the dorsal hippocampus (dentate gyrus (DG), CA1 and CA3 areas), and the perirhinal (PRh) and entorhinal (Ent) cortices. Data were analyzed using one-way ANOVA followed by Tukey’s test (*#&*p*< .05; * HFHC+PBS vs. HFHC+LGG and HFHC+AKK vs. NC; # HFHC+LGG and HFHC+AKK vs. HFHC+PBS; and HFHC+AKK vs. HFHC+LGG)

Region	NC	HFHC+PBS	HFHC+LGG	HFHC+AKK
**PrL**	28.526 ± 1.327	* 22.244 ± 1.336	* 20.738 ± 0.403	& 25.929 ± 1.079
**IL**	28.153 ± 1.109	* 21.897 ± 0.998	* 19.785 ± 0.510	* & 24.277 ± 1.112
**Cg**	28.846 ± 1.333	* 22.650 ± 0.991	* 22.101 ± 0.545	& 26.131 ± 1.077
**dST**	28.040 ± 0.707	* 22.484 ± 0.878	* 22.844 ± 0.473	#& 26.125 ± 0.775
**AcbC**	35.734 ± 1.337	* 26.777 ± 0.920	* 24.850 ± 0.786	* 28.662 ± 1.185
**AcbSh**	39.684 ± 1.055	* 30.770 ± 1.405	* 29.465 ± 1.298	* 31.602 ± 1.731
**AMT**	26.087 ± 1.280	22.187 ± 0.556	* 21.189 ± 0.815	23.501 ± 0.897
**ADT**	37.683 ± 1.684	35.689 ± 1.132	34.485 ± 0.999	37.131 ± 0.992
**AVT**	31.661 ± 1.702	27.654 ± 1.206	27.254 ± 0.856	29.324 ± 0.708
**CeA**	23.299 ± 0.984	* 18.874 ± 0.849	20.607 ± 0.456	# 22.133 ± 0.827
**BLA**	25.912 ± 1.338	* 21.297 ± 1.131	22.484 ± 0.400	·# 25.123 ± 0.628
**LaA**	20.135 ± 0.828	18.128 ± 0.688	19.400 ± 0.342	20.002 ± 0.889
**DG**	34.185 ± 1.729	* 28.133 ± 1.170	* 25.733 ± 0.726	* 29.408 ± 0.964
**CA1**	20.905 ± 1.154	* 17.692 ± 0.686	* 16.905 ± 0.442	17.929 ± 0.695
**CA3**	20.556 ± 1.127	* 16.890 ± 0.274	* 16.615 ± 0.237	17.787 ± 0.669
**PRh**	25.015 ± 1.240	*20.272 ± 0.405	* 18.797 ± 0.554	19.835 ± 0.510
**Ent**	21.212 ± 1.502	17.752 ± 0.405	17.348 ± 0.368	18.844 ± 1.051

When the animals were treated with *A. muciniphila* CIP107961, their brain metabolic activity equaled that of the NC group in most of the regions previously affected by HFHC, such as the prefrontal cortex, dorsal striatum, amygdala, hippocampus, and perirhinal cortex. In line with this, HFHC+AKK showed an increased CCO value in the dorsal striatum (dST, *p*< .001), central amygdala (CeA, *p*= .004), and basolateral amygdala (BLA, *p*= .014), compared to HFHC+PBS. However, the HFHC+AKK group also showed lower CCO levels than the NC group in the infralimbic cortex (IL, *p*< .001), ventral striatum (*p*< .001), and dentate gyrus (DG, *p*< .001).

Regarding *L. rhamnosus* GG administration, we found that the HFHC+LGG group maintained decreased levels of CCO, compared to the NC group, in the prefrontal cortex (*p*< .001), dorsal and ventral striatum (*p*< .001), thalamic nuclei, such as anteromedial nucleus (AMT, *p*= .016), hippocampus (*p*≤ 0.007), and perirhinal cortex (PRh, *p*< .001). More importantly, HFHC+LGG did not display significant differences in CCO levels compared to HFHC+PBS.

When comparing the two probiotic treatments, we observed that the HFHC+AKK group showed statistically significant higher CCO values than the HFHC+LGG group in the prefrontal cortex (*p*< .001) and dST (*p*< .001). These results indicate that the decreased CCO shown by HFHC+PBS cannot be recovered with LGG administration, whereas the opposite effect was found when applying AKK. More importantly, *A. muciniphila* CIP107961 was able to reverse the HFHC-associated decrease in CCO activity in most of the brain regions previously affected by the HFHC diet.

### Probiotics do not induce major rearrangements in the fecal microbiota

**Figure 4.**
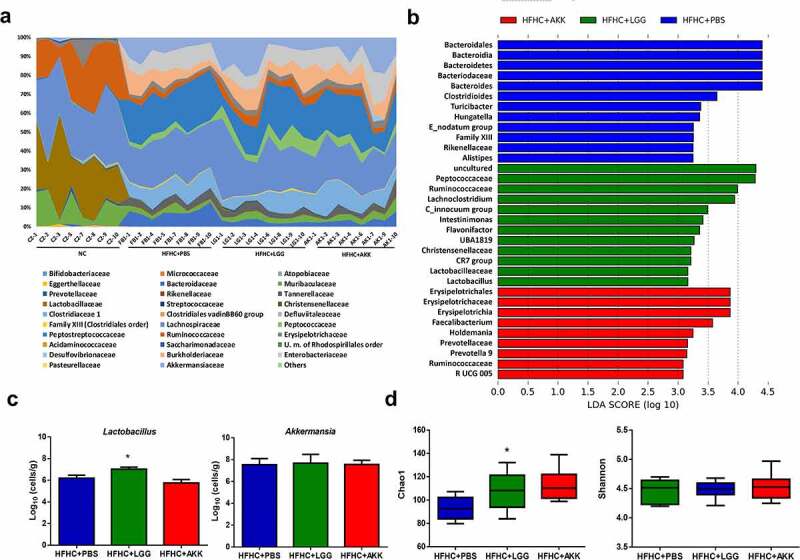
Gut microbiota exploration in experimental groups subjected to PBS, LGG, and AKK. (a) **Aggregate microbiota composition**. Average relative abundance of gut microbiota at the family level from NC, HFHC+PBS, HFHC+LGG, and HFHC+AKK groups. Bacterial taxa representing less than 0.5% of the total abundance are included in Others. (b) **Gut microbiota differences**. Results of LEfSe analysis (LDA scores >2 and significance of *p*< .05 as determined by Wilcoxon’s signed-rank test) showing significantly different taxa among the HFHC+PBS, HFHC+LGG, HFHC+AKK groups. Red indicates differential abundance in the HFHC+AKK group; green indicates differential abundance in the HFHC+LGG group; blue indicates differential abundance in the NC+PBS group. Bacterial taxa representing less than 0.5% of the total abundance are included in Others. (c) **qPCR concentration**. Bar charts (mean ± SEM) represent comparison of *Lactobacillus* and *Akkermansia* genus concentration analyzed by qPCR (log_10_ cells/g feces), compared using the Kruskal–Wallis test followed by Dunn’s analysis (*p < .05, comparison with all the groups). (d) **Bacterial diversity**. Box and whiskers (median and IRQ range) represent comparison of alpha-diversity of gut microbiota using Chao1 and Shannon indexes among groups, compared using a one-way ANOVA followed by Tukey’s test (*p < .05, comparison with HFHC+PBS group)

To assess the effect of two different probiotics on the NASH-associated cognitive disturbances, we first analyzed the effect of the administration of the strains by oral gavage on the gut microbiota in the gavage feeding groups (HFHC+AKK, HFHC+LGG, and HFHC+PBS (control group)), also including the NC group (no gavage) as external control. MiSeq sequencing produced an average of ~63,000 filtered partial sequences per sample, and it showed the biggest differences at the diversity and compositional levels between the NC group and the other three NASH groups (Figure 4(a); Supplemental Figure 3(a)), confirming the strong effect of diet (control vs. HFHC diet). The analyses of the SCFAs followed the same trend (Supplemental Figure 3(b)). These results are in concordance with our previous observations in the NASH^3^ animal model. Thus, by focusing more on the specific effect of the two probiotics on the gut microbiota, we observed that the administration of both bacteria led to a unique change in the microbial composition at the phylum level, by significantly decreasing Bacteroidetes, compared to placebo administration (PBS) (*p = *.016 HFHC+LGG vs. HFHC+PBS; *p = *.018 HFHC+AKK vs. HFHC+PBS). At lower taxonomical levels, applying a linear discriminant analysis effect size (LEfSe) method, we observed that only a few families and genus suffered differential changes in their abundance depending on the probiotic administration. LGG produces, among others, a statistically significant increase in the relative abundance of *Christensenellaceae, Ruminococcaceae, Peptococcaceae*, and *Lactobacillus* (Figure 4(b)). In the HFHC+AKK group, we observed higher abundance, mainly in the *Faecalibacterium, Prevotella* 9, and the *Ruminococcus* UCG005 group (Figure 4(b)). Changes in the concentration of the *Lactobacillus* and *Akkermansia* genus were validated by qPCR, confirming higher levels of the *Lactobacillus* genus in the HFHC+LGG group and no differences in the *Akkermansia* genus across the groups (Figure 4(c)). In contrast, the Chao1 index showed statistically higher alpha-diversity in the HFHC+AKK group compared to the HFHC+PBS group (*p < *.05); however, the Shannon index did not show any significant differences among the groups (Figure 4(d)). These results indicate that probiotics can affect microbial diversity, but they did not induce major rearrangements of the fecal microbiota.

Finally, the levels of the main SCFAs did not show differences between the probiotic groups and the HFHC+PBS group, with the exception of acetate, which was lower in the probiotic groups (*p < *.01, HFHC+LGG vs. HFHC+PBS; *p < *.05, HFHC+AKK vs. HFHC+PBS) (Supplemental Figure 3(c)).

## Discussion

In this study, we described cognitive, brain metabolism, and microbiota alterations associated with high-fat and high-cholesterol consumption. In addition, we clearly showed that environmental enrichment and *A. muciniphila* CIP107961 restore cognitive dysfunction. Furthermore, we revealed that cognitive improvement is associated with differential effects of environmental enrichment and this strain of *A. muciniphila* on brain metabolism and gut microbiota. Finally, we discovered that restored cognitive function was associated with the administration of *A. muciniphila* CIP107961, but not *L. rhamnosus* GG, which may be clinically relevant when selecting probiotics for treating HFHC-derived pathologies.

Environmental enrichment (EE) has been described as motor and sensorial stimulation that is able to modulate the physical and cognitive status.^23^ Moreover, EE has been found to have brain repairing therapeutic effects in several neuropsychiatric diseases.^22–24^ Our results revealed that the HFHC group presented cognitive deficits, which are in line with previous studies where impaired cognitive function has been associated with high-fat diet consumption and alterations of gut microbiota.^31,32^ Specifically, novel object recognition impairment was restored by EE, in accordance with de Souza et al.^33^ Our data extend these findings, demonstrating that spatial working memory alterations, which we have previously described,^3^ were also improved by EE. The effect of EE on the HFHC group was accompanied by decreased levels of brain metabolism in the prefrontal cortex, striatum, and perirhinal cortex. Moreover, other brain regions, such as the thalamus, the amygdala, and the hippocampus, showed the same pattern. The prefrontal cortex sends projections to the perirhinal and entorhinal cortices^34^ and is in reciprocal connection with the striatum^35^ and the thalamus.^36^ This network supports spatial working memory and novel object recognition abilities.^34,36–38^ Therefore, any improvement in this network will be reflected in an improvement in both behavioral tasks, as we demonstrated.

One additional finding is the decrease in brain metabolic levels when introducing EE, which was clearly observed not only in the HFHC+EE group but also in the NC+EE group. Decreased brain CCO activity after EE has been previously reported by several authors in rats and mice, and may be related to higher metabolic efficiency requiring less energy demands by brain cells to perform an improved behavioral response^39,40^ by comparison to the HFHC condition. These results are in line with previous data by Tong et al.^41^ showing that exercise decreased the hippocampal expression of genes involved in mitochondrial metabolic processes, including cytochrome c-oxidase. In fact, physical activity and EE have been found to regulate metabolic and redox activity, resulting in beneficial modulation of brain function.^42^ Similarly, our results support the overall effect of EE on brain metabolism through the reduction in metabolic levels in both healthy and HFHC conditions. However, a better understanding of the EE-induced brain network modifications could be a key factor to understand mechanisms of memory processes and thus would help to develop new therapeutic strategies to alleviate memory deficits such as those related to HFHC brain dysfunction.

When we assessed the gut microbiota composition after EE implementation, we showed an increase in the bacterial richness in the NC+EE group. Moreover, EE induced modifications at the taxonomical level in the HFHC group, decreasing Proteobacteria and increasing Firmicutes. Exercise, which could be understood as EE, has been demonstrated to modulate gut microbial composition and diversity in both the short-^43^ and long-term.^44,45^ Choi et al.^46^ showed that when physical exercise was performed by mice, they showed greater abundance of the Lactobacillales order and more Enterococcus faecium bacteria than sedentary mice. Similar results were presented by Queipo-Ortuno et al (2013),^47^ in which increased *Lactobacillus* and *Blautia coccoides–Eubacterium rectale* groups were found in the exercised rats. Furthermore, in a study carried out with diverse rat strains, an increase in bacterial diversity in exercised rats was described, and more specifically an increase in the *Lactobacillus* genus in obese rats subjected to physical exercise.^48^

Regarding the effect of the exercise on microbiota changes induced by the diet, Kang et al.^49^ proved that exercise not only counteracted the microbiota changes induced by the high-fat diet but caused large shifts in Firmicutes, Bacteroidetes, and Tenericutes phyla in the same direction and order of magnitude as those caused by the high-fat diet. Our results could be in line with these studies due to the fact that increased abundance of Firmicutes phyla and *Akkermansiaceae* has been shown in the HFHC+EE group, whereas increased abundance of *Micrococcaceae, Christensenellaceae*, and *Ruminococcaceae* have been found in the NC+EE group. These data may indicate that changes induced by exercise are influenced by the metabolic state of the individuals, and this factor should be taken into account in further translational studies.

Gut microbiota changes provoke a dysregulation of the enteric nervous system, which leads to a breakage of the gut–brain axis and ultimately to neural deficits.^50,51^ However, although the mechanisms by which exercise causes changes in the microbiota are not fully understood, there are probably several factors and pathways involved.^52^ Our results did not show significant changes in SCFAs in the groups exposed to EE (NC+EE and HFHC+EE), although decreased iso-butyrate levels were found in the HFHC+EE group. The fact that EE did not affect SCFAs in any of the conditions (NC and HFHC) but reverses the effects on learning ability indicates only that 4 weeks exercise could mediate cognitive improvements through other mechanisms (see Kempermann (2019)),^53^ which is in contrast to SCFAs. In addition, gut dysbiosis has been associated with mitochondrial dysfunction through the delivery of metabolites that can affect reactive oxygen species and ATP production,^50^ suggesting a potential mechanism for neural damage. The NC, NC+EE, and HFHC+EE groups did not show the cognitive impairment displayed by the HFHC group; they manifested a better capability for novel object recognition and spatial working memory. Hence, these results demonstrated that EE is able to target the gut–brain axis, leading to the modulation of brain oxidative metabolism, which has an impact on cognitive amelioration.

Regarding probiotic administration, we found that LGG was not effective in ameliorating the cognitive dysfunction in the HFHC group, showing similar results to those found in the control group (HFHC+PBS). These results are in line with a previous study by Kelly et al.^54^ in healthy humans, where LGG was not able to improve cognitive function, measured as visuospatial memory performance, attention switching, rapid visual information processing, and emotion recognition. At the compositional level, the gut microbiota of the HFHC+LGG group suffered slight modifications with regard to the placebo group (HFHC+PBS), with a considerable increase in *Lactobacilli* and a lower concentration of acetate in fecal water. In contrast to the results observed with LGG, the HFHC animals receiving *A. muciniphila* CIP107961 displayed correct performance in novel object recognition and spatial working memory, which means that this microorganism is effective in reversing HFHC-associated cognitive defects.

Supporting these results, other studies have found that NASH animals displayed behavioral abnormalities that have been linked to an increased protein expression of Toll-like receptor 4 (TLR4), a major regulator of pro-inflammatory response and microglial activation, in the prefrontal cortex but not elsewhere.^55,56^ Moreover, in NASH animals, signs of microglial activation and oxidative stress in the prefrontal cortex have been found^57^ together with increased protein oxidation and lipid peroxidation in the frontal cortex.^58^ *A. muciniphila* has been shown to reduce microgliosis and inflammation, and to rescue hippocampal-dependent cognitive function in HF diet-fed mice.^59^

In addition, this cognitive improvement could be explained by the effect of *A. muciniphila* on glucose levels,^60^ which can also support not only restored metabolic levels in the amygdala and the dorsal striatum, but also the higher CCO values found in the prefrontal cortex compared to HFHC+LGG in this latter area. In this regard, we also found that *A. muciniphila* CIP107961 displayed similar values to the NC group in regions previously affected by HFHC, such as the prefrontal cortex, striatum, hippocampus, and perirhinal cortex. When we addressed the *L. rhamnosus* GG administration, no changes were found in the CCO values in any of the measured regions compared to HFHC+PBS, which is consistent with the inability of this group to improve their cognitive function.

An interesting finding of our study is the decreased CCO levels when comparing the HFHC+PBS and NC groups in the prefrontal, hippocampus, amygdala, and perirhinal cortices. These results could reflect a stress response^61,62^ potentially induced by gavage administration.^63^ The gut microbiota study after *A. muciniphila* CIP107961 administration did not show major rearrangements, with only slight modifications and a modest increase in bacterial diversity compared with the microbiota of animals receiving PBS or LGG. Similar results were obtained by Yang et al.^59^ and it could suggest that major microbiota changes are not involved in the observed effects, which is in line with previous studies carried out in other animal models^28,64^ or in humans^30^ that reported beneficial effects of these microorganisms without observing major changes in the gut microbiota composition. However, it is important to note that more in-depth studies may be needed to fully elucidate this issue because in this study we rely on 16S rRNA gene-based microbiota profiling, and, therefore, we do not have a complete overview of the total intestinal metagenome.

We finally wanted to compare the two successful treatments, environmental enrichment and *A. muciniphila* CIP107961 administration, to elucidate their differential effects along the gut–brain axis. We first showed that each treatment produces an opposite effect on brain metabolism: whereas EE caused an overall brain metabolic decrease, AKK restored the activity. Second, in a similar manner, HFHC+EE and HFHC+AKK animals showed clear differences in their microbiota patterns. Whereas the HFHC+AKK group showed only minor differences with regard to the corresponding experimental control group (HFHC+PBS), the differences with the HFHC+EE group were larger, including higher levels of Bacteroidetes and Proteobacteria at the phylum level. However, as previously discussed, the existence of differences in the basal microbiota between these two groups prevents us from drawing firm conclusions. Indeed, these differences suggest that the observed effects of both EE and *A. muciniphila* administration on the reversal of NASH-associated cognitive impairments are independent from a defined overall microbiota composition. In this regard, some studies have identified specific *A. muciniphila* proteins, such as the membrane protein Amuc_1100, as being able to mediate some of these beneficial effects.^65^

Finally, we want to point out the observed changes in the SCFAs and their metabolites, which showed a decrease in iso-butyrate and acetate when the animals were exposed to environmental enrichment or treated with probiotics, respectively. In this regard, some microorganisms, such as Bacteroides or Clostridia members, are able to produce BCFAs from the proteolytic metabolism of branched-chain amino acids. The higher levels of *Bacteroidaceae* or *Peptostreptococcaceae* observed in the HFHC group could be related to the higher levels of BCFAs observed, as found in other diseases related to the gut–brain axis, such as obesity,^66^ Rett syndrome,^67^ or anorexia nervosa.^68^ Furthermore, evidence exists in mice that acetate can alter the levels of glutamate, glutamine, and GABA,^69^ and some studies have shown that SCFAs and their metabolites can stimulate vagus nerve signaling.^8,9^ Moreover, other studies have correlated changes in SCFAs and iso-butyrate with positive effects on behavior through the microbiota–gut–brain axis.^7^ Hence, these results could highlight the contribution of SCFAs and BCFAs to NASH-cognitive improvement.

In conclusion, the microbiota and cognition are intimately connected through the gut–brain axis, and in HFHC pathologies they can be influenced by environmental enrichment and *A. muciniphila* CIP107961 administration. Cognitive improvement was accompanied by changes in brain metabolic activity and gut microbial composition analysis, pointing to specific microbiota targets for intervention in diet-induced pathologies. However, some mechanisms other than major changes in microbiota composition and the combined effect of environmental enrichment and *A. muciniphila* administration, which we identified in this study, may also be biologically relevant and will need to be investigated in future studies due to their relative contributions to the selection of effective treatments for patients.

## Material and methods

### Experimental groups

Fifty-six male Sprague-Dawley rats (220 g at the start) (Envigo, United Kingdom) were divided into seven groups (n = 8 per experimental group): NC (normal chow), NC+EE (normal chow + environmental enrichment), HFHC (high-fat, high cholesterol diet), HFHC+EE, HFHC+PBS (phosphate-buffered saline), HFHC+LGG (*Lacticaseibacillus rhamnosus* GG, ATCC53103), and HFHC+AKK (*Akkermansia muciniphila* CIP107961). Normal chow contained 13 kcal% from fat and no cholesterol (Envigo, United Kingdom – 2914), and NASH was induced through a HFHC diet (Research Diets, USA – D09052204) containing 65 kcal% from fat and 2 kcal% cholesterol. All the groups were weighed weekly and studied after 14 weeks of the administration of the diet (Figure 1(a)). Each subject was subjected to a different intervention and performed the behavioral tests only once. The animals had ad libitum tap water and were maintained at constant room temperature (22 ± 2°C), with a relative humidity of 65 ± 5% and an artificial light/dark cycle of 12 h (08:00–20:00/20:00–08:00 h).

The procedures and manipulation of the animals used in this study were carried out according to the Directive (2010/63/EU), Royal Decree 53/2013 of the Ministry of the Presidency related to the protection of animals used for experimentation and other scientific purposes.

### Environmental enrichment

The NC+EE and HFHC+EE groups were continuously submitted to EE from week 8 to week 12 (Figure 1(a)). During this time, each experimental group was separately housed in groups of 8 subjects in large cages measuring 76.5 × 48 × 81 cm, maintaining the same previous contingencies in terms of food, water administration, and room conditions. The cages contained different stimulating objects, including platforms, tubes, little houses, running wheels, balls, and toys made of different materials, textures, shapes, sizes, and colors.^70^ The stimulating objects were the same in both groups, and they were changed weekly to ensure novelty.

### Probiotics culture and administration

Two different bacterial strains were used, *L. rhamnosus* GG (ATCC 53103) (LGG) and *A. muciniphila* CIP107961 (AKK). *L. rhamnosus* GG was grown in a de Man, Rogosa and Sharpe (MRS) medium (Difco, Becton Dickinson), and *A. muciniphila* CIP107961 was grown in a GAM medium (Nissui Pharmaceutical Co.). Both were incubated at 37°C in an anaerobic chamber (Mac 500; Don Whitley Scientific) under a 10% (v/v) H_2_, 10% (v/v) CO_2_ and 80% (v/v) N_2_ atmosphere. Twenty-four hour culture was used to inoculate (1% v/v) fresh MRS broth or a pre-reduced GAM broth medium, which were incubated overnight (*L. rhamnosus* GG) and for 24 h (*A. muciniphila* CIP107961). Then, the cultures were washed and concentrated with pre-reduced PBS, which included 20% (v/v) glycerol to a concentration of about 1 × 10^10^ cfu/ml and was stored at −80°C until administration. The HFHC+LGG and HFHC+AKK animals were administered their respective bacteria at a final concentration of 1 × 10^9^ cfu. Before gavage, glycerol stocks were thawed under anaerobic conditions, centrifuged to remove glycerol, and resuspended in 1 ml of pre-reduced PBS. HFHC+LGG and HFHC+AKK rats were administered 100 μl of *L. rhamnosus* GG and *A. muciniphila* CIP107961, respectively, and a control group received 100 μl of PBS (HFHC+PBS) daily from weeks 8 to 12 of the HFHC diet (Figure 3(a)).

Viability of the glycerol stocks was tested by serial dilutions in PBS and plating counts in MRS (48 h) and GAM-agar (5 days), respectively, and incubation under anaerobic conditions. Both strains were validated for purity by whole-gene 16S Sanger sequencing.

### Fecal sample collection and processing

Fresh fecal pellets from each animal were collected and kept at −80°C until DNA extraction. Before that, samples were weighed, diluted in PBS (1:5 w/v), and homogenized (3 min, full speed) in a stomacher (LabBlender 400). From 1 mL of the homogenate, fecal supernatant and cellular pellets were separated by centrifugation (10,000 rpm, 15 min) and stored at −20°C. The supernatant was used for SCFA and BCFA analysis, and the pellets were used for DNA extraction with the QIAamp DNA stool kit (Qiagen, GmbH, Germany), as described elsewhere.^71^

### Analysis of fecal microbial groups by 16S rRNA gene profiling and quantitative PCR

Extracted Isolated DNA was used as a template for amplification of the V3 region from partial 16S rRNA gene sequences by PCR, using the primers and conditions described by Milani and coworkers.^72^ The amplicons obtained were then sequenced by using the MiSeq (Illumina) platform) at GenProbio srl (Italy). The individual reads obtained were filtered, trimmed, and processed.^73^ 16S rRNA Operational Taxonomic Units were defined at ≥97% sequence homology using the uclust tool developed by Edgar.^74^ All reads were classified in the lowest possible taxonomic rank using QIIME 2 and a reference dataset from the SILVA database.^75^ *Akkermansia* and *Lactobacillus* genus were also determined by quantitative PCR using the previously described primers and conditions.^76^

### Determination of short-chain and branched-chain fatty acids in feces

SCFA and BCFA levels were determined in the fecal supernatants by means of gas chromatography, as described by Moris et al.^77^ Briefly, 250 μl of cell free-supernatants were mixed with 100 μl methanol, 50 μl internal standard solution (2-ethylbutyric 1.05 mg/ml), and 50 μl of 20% v/v formic acid. The mix was then centrifuged, and the supernatant was injected into a system composed of a 6890NGC injection module (Agilent Technologies Inc., USA) connected to a flame injection detector (FID) and a mass spectrometry detector (MS, 5973 N) (Agilent) for quantification of both SCFA and BCFA.

### Locomotor activity assessment

The study of locomotor activity was carried out using a Rotarod 7750 for rats, which consists of a motor-driven rotating rod (Ugo Basile Biological Research Apparatus).^78^ After habituation for 1 min at a constant speed of 2 rpm, the rats were evaluated for 5 min, during which the speed increased constantly until 20 rpm. Rotation speed was recorded and used as a measure of locomotor function.

### Novel object recognition

The object recognition test was carried out in an open field (66 × 46 × 45 cm) made of gray fiberglass, with an open roof. The open field was situated in a room with two diffuse white lights on its sides, providing an illumination density of 50 lux at its center. The animal’s behavior was recorded by a video camera (Sony V88E) connected to a computer equipped with a computerized video-tracking system (EthoVision Pro, Noldus Information Technologies, The Netherlands). After each trial, the apparatus was thoroughly cleaned with a 75% ethanol solution. The objects used were constructed from a combination of plastic pieces of different colors and shapes. The animals were habituated for two days before the test. On the first day, the animals were situated first in groups and then individually in the open field for two trials lasting 6 minutes each. On the second day, each animal was placed in the field, with two equal objects in the center, for 3 trials lasting 6 minutes each, separated by 20 minutes.

The test was composed of two phases. In the first, in the open field, each subject was exposed for 4 minutes to two copies of a new object (2 × object A), located in opposite corners at 10 cm from the walls. After a 50-minute delay, each animal carried out the second trial, which was similar to the previous one, except that a copy of the previously-encountered object was changed to a novel one (object A + object B).

Exploration of an object was defined as directing the nose toward the object at less than 2 cm and exploring it (i.e., sniffing and or interacting with the object). For each animal, time spent exploring the objects in both trials (acquisition/sample and retention/test) was measured. The exploratory preference during the acquisition/sample (E1) and retention/test (E2) phases of the test was considered as the ratio between the exploration of the objects in each phase and the total time available for exploration. The discrimination of the objects was assessed through the discrimination index (D1), the difference in time spent exploring the two objects in the second phase (novel minus familiar), and the discrimination ratio (D2), the difference in time spent exploring the two objects in the second phase divided by the total time exploring both objects.^37^

### Spatial working memory

Spatial working memory was measured in the Morris water maze (MWM), which is a cylindrical fiberglass tank, virtually divided into four quadrants, measuring 150 cm in diameter with a 40 cm high wall.^79^ The water level was 30 cm, and its temperature was 22 ± 2°C. The pool contained a cylindrical platform that was 10 cm in diameter and 28 cm high, of which 2 cm was below the surface and used by the animals to escape. The MWM was in the center of a 16 m^2^ lit room (two halogen lamps of 4000 lx), surrounded by panels on which several extra-maze clues were placed. The animal’s behavior was recorded by a video camera (Sony V88E) connected to a computer equipped with a computerized video-tracking system (EthoVision Pro, Noldus Information Technologies, The Netherlands). One day before the test, the animals were habituated to the task in 3 trials with the platform, using different starting positions in a small square water tank (47 × 75 × 38 cm).

The spatial working memory test was a paired sample task carried out for five days. Each session consisted of two trials (sample and retention). Within the same session, the platform, which was invisible to the animals because it was hidden underwater, was situated in one of the four quadrants and the animal started facing the wall of another quadrant. The trial ended once the animal had found the platform, or when 60 s had elapsed. If the animal had not reached the hidden platform after this time, it was placed on the platform for 15 s. During the intertrial interval, the animals were placed in a bucket for 5 s. The quadrant containing the platform and the animal’s starting point changed between sessions. Latencies to reach the escape platform were recorded and used as a measure of task acquisition. The learning criterion was when the animals spent significantly less time in the retention trial than in the sample trial in each session.^80^

### Brain metabolic activity

Ninety minutes after the last session of the spatial working memory task, the animals were decapitated, their brains were frozen and subsequently sliced in 30 µm-thick brain coronal sections. The protocol followed was previously described by our group,^81^ and staining variability across different baths was controlled through sets of brain tissue homogenate standards of known CCO activity from rat brains cut at different thicknesses (10, 30, 50, and 70 µm).^82^

The CCO histochemical staining intensity was quantified by densitometric analysis, using a computer-assisted image analysis workstation (MCID, Interfocus Imaging Ltd). The mean optical density (OD) of each region was measured in twelve readings that were averaged to obtain one mean per region for each animal. These OD values were then converted to CCO activity units (µmol of cytochrome c oxidized/min/g tissue wet weight), determined by the enzymatic activity of the standards measured spectrophotometrically.

Neuronal metabolic activity was measured in selected brain regions anatomically defined according to Paxinos and Watson’s atlas.^83^ The regions of interest and their distance from bregma were: the prefrontal cortex (+3.24 mm) (prelimbic (PrL), infralimbic (IL) and cingulate (Cg) cortices), the dorsal striatum (dST) (+1.56 mm) and ventral striatum (+1.56 mm) (accumbens core (AcbC) and shell (AcbSh)), the thalamus (−1.20 mm) (anteromedial nucleus (AMT), anterodorsal nucleus (ADT), and anteroventral nucleus (AVT)), the amygdala (−2.28 mm) (central (CeA), basolateral (BLA) and lateral (LaA)), the dorsal hippocampus (−3.00 mm) (dentate gyrus (DG), CA1 and CA3 areas), and the perirhinal (PRh) and entorhinal (Ent) cortices (−4.20 mm).

### Statistical analysis

Data derived from microbiota were analyzed using the IBM SPSS Statistics Version 24.0 (IBM Corp., USA) software. Comparisons of the groups following normal distributions (alpha-diversity) were performed using one-way ANOVAs, followed by Tukey’s post-hoc analyses. For non-parametric distributions (qPCR, SCFA, and BCFA data), the Kruskal–Wallis test was performed, followed by Dunn’s post-hoc test. Differences in bacteria abundances were calculated using the linear discriminant analysis effect size (LEfSe) method.^84^

Body weight, behavioral data, and CCO values were analyzed with the SigmaStat 3.2 program (Systat, USA). Body weight was analyzed using a two-way ANOVA (Group × Week), followed by a post-hoc Tukey test when appropriate. Maximum speed spent on the Rotarod was compared between groups using a Kruskal–Wallis test. In the novel object recognition test, two-way ANOVA (Diet × Treatment) was used to compare exploration and discrimination index and ratio followed by a post-hoc Tukey test when appropriate. In the spatial working memory test, sample and retention trial latencies were compared within each group with a two-tailed paired *t*-test, and between groups with a one-way ANOVA. Brain metabolic activity was evaluated between the groups through one-way ANOVA. When normality or equal group variances failed, Kruskal–Wallis one-way analysis of variance on ranks was performed. Post-hoc multiple comparison analyses were carried out, when allowed, using the Tukey method. The results were considered statistically significant if *p*< .05.

## Supplementary Material

Supplemental Material
