# Supplementary material for: Akkermansia muciniphila and environmental enrichment reverse cognitive impairment associated with high-fat high-cholesterol consumption in rats
Source: Gut Microbes. 2021 Mar 8;13(1):1880240. doi: 10.1080/19490976.2021.1880240 (PMC7946069; doi:10.1080/19490976.2021.1880240)
Supplement: Supplemental Material [file KGMI_A_1880240_SM4640.docx]

**Supplemental Figures and Figure legends**


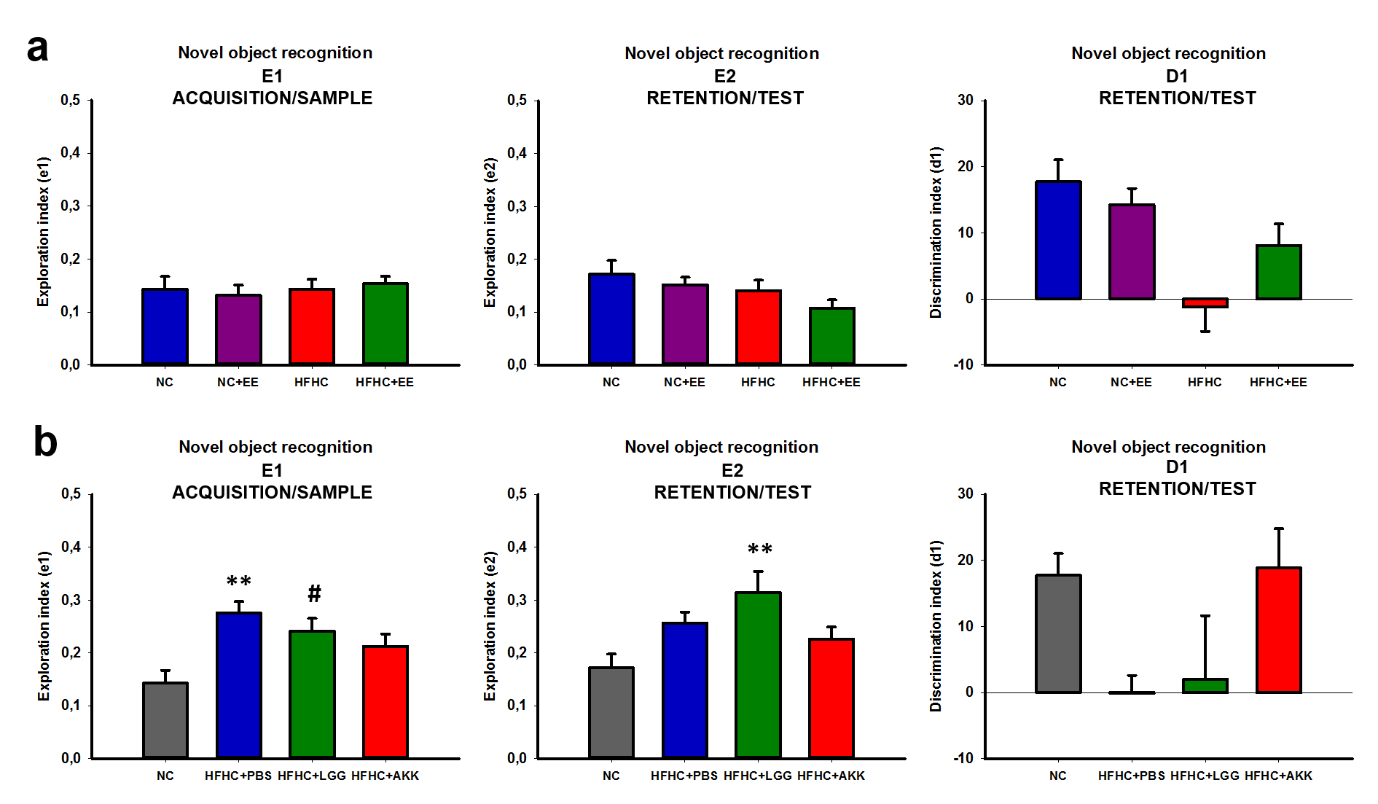


***Supplemental figure 1. Indexes of exploration and discrimination of the objects.* (a)** Bar charts (mean±SEM) represent exploratory indexes in acquisition/sample (E1) and retention/test (E2) phases and discrimination index (D1) during retention/test phase between NC, NC+EE, HFHC, HFHC+EE groups. These values were compared through a two-way ANOVA followed by Tukey’s post-hoc analyses (#*p*<0.05, ***p*≤0.010). **(b)** Bar charts (mean±SEM) represent exploratory indexes in acquisition/sample (E1) and retention/test (E2) phases and discrimination index (D1) during retention/test phase between NC, HFHC+PBS, HFHC+LGG, HFHC+AKK groups.


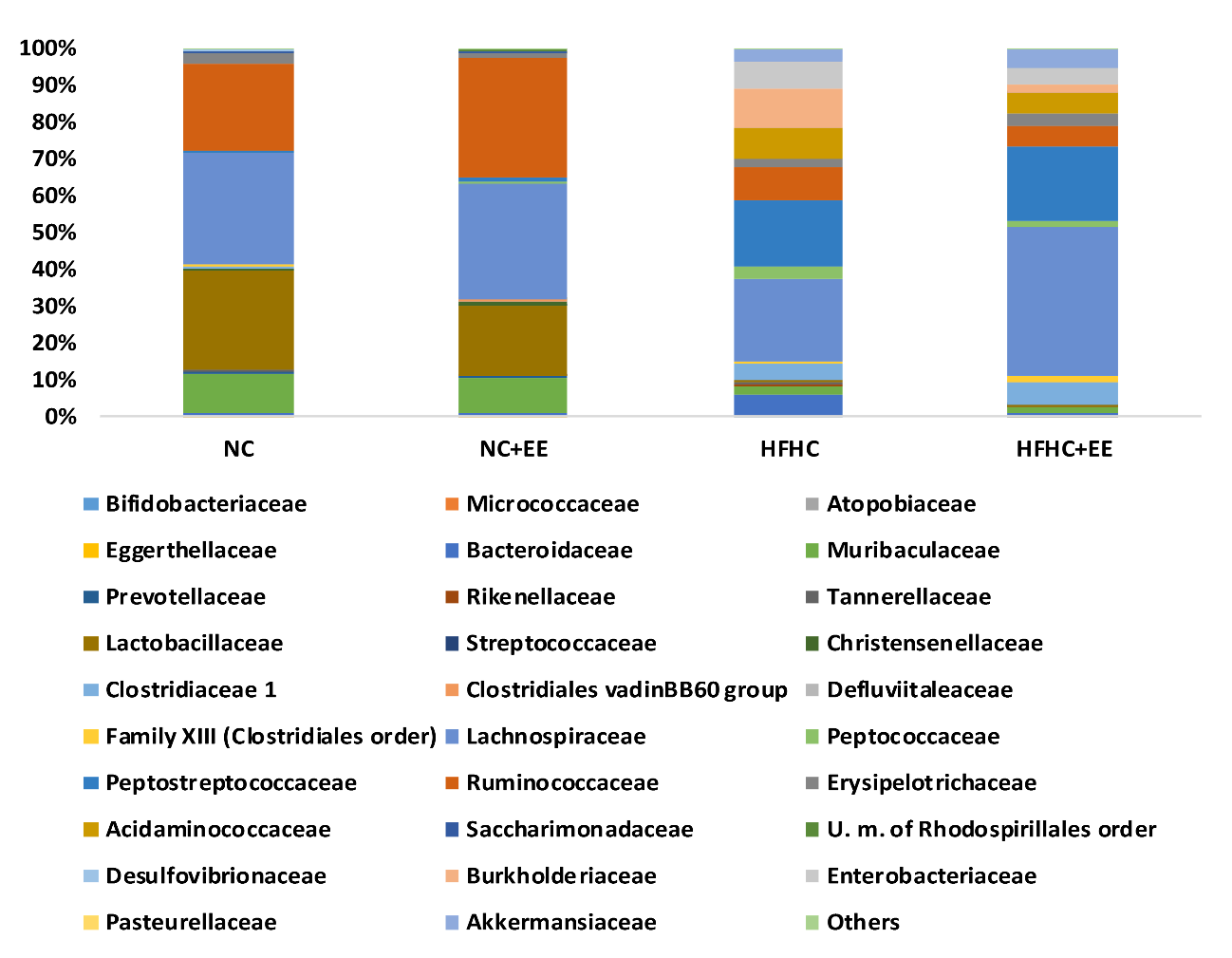


***Supplemental figure 2. Gut microbiota assessment of different groups subjected to NC and HFHC diets and the implementation of EE*.** Average relative abundance of prevalent microbiota at the family level from NC, NE+EE, HFHC, HFHC+EE groups. Bacterial taxa representing less than 0.5% of the total abundance are included in Others.

**
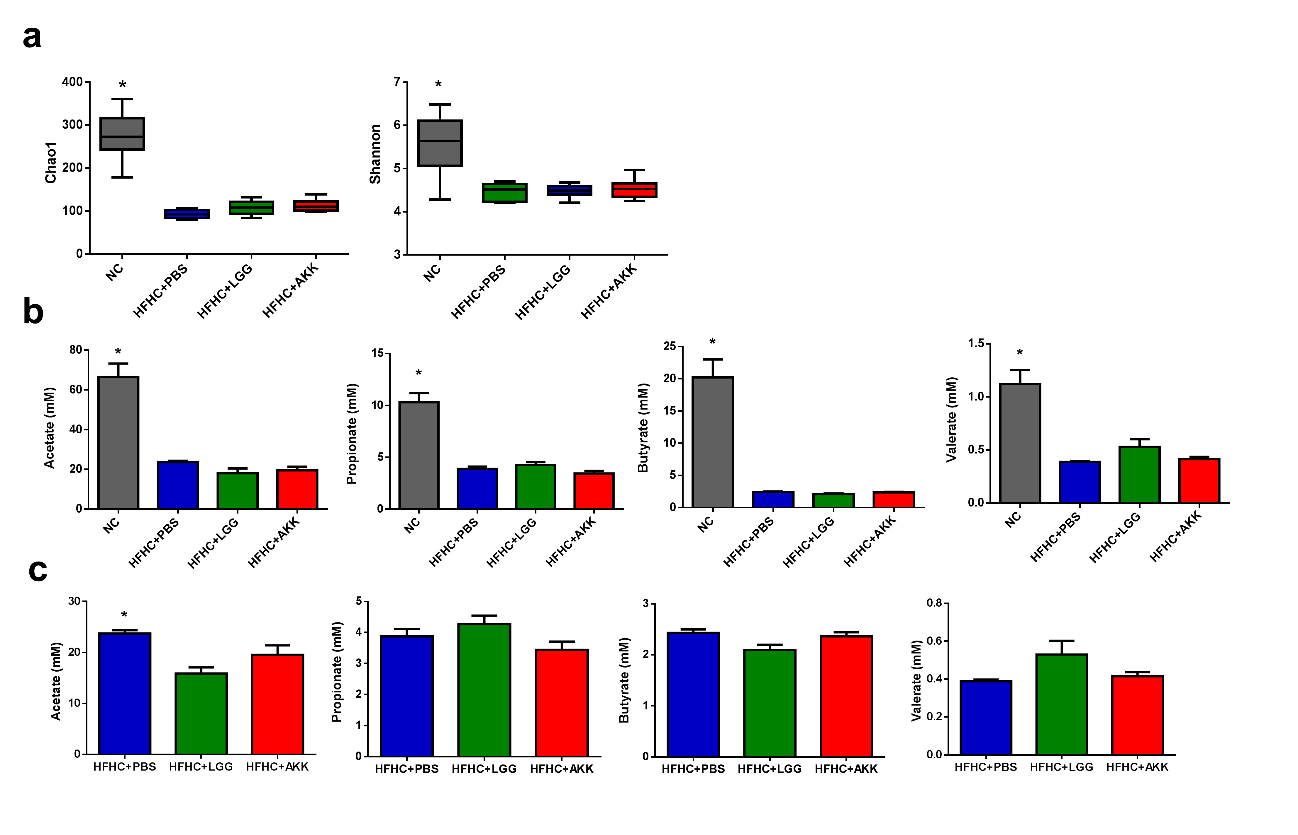
**

***Supplemental figure 3. Gut microbiota analysis in different groups subjected to NC and HFHC diets and the administration of PBS, LGG and AKK.*** **(a) Bacterial diversity**. Box-and-whiskers (median and IRQ range) represent comparison of alpha-diversity of gut microbiota using Chao1 and Shannon indexes among the groups studied, compared through one-way ANOVA followed by Tukey’s test (* comparison with all the groups). **(b) SCFAs when comparing NC and HFHC groups**. Bar charts (mean±SEM) represent comparison of the SCFAs levels (mM) compared using the Kruskal-Wallis test followed by Dunn’s analysis (* comparison with all the groups). **(c)** **SCFAs when comparing experimental HFHC groups**. Bar charts (mean±SEM) represent comparison of the SCFAs levels (mM), compared using the Kruskal-Wallis test followed by Dunn’s analysis (* comparison with all the groups). **p<*0.05.

**
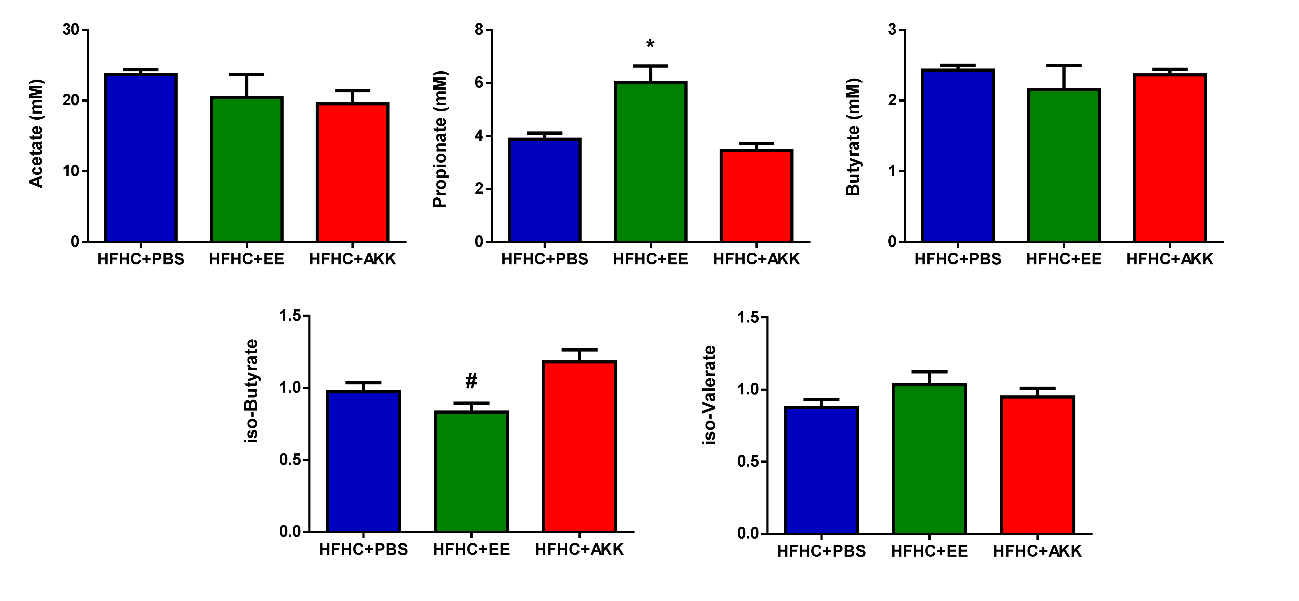
**

***Supplemental figure 4. SCFAs analysis in HFHC groups subjected to the administration of PBS, AKK and EE.*** Bar charts (mean±SEM) represent comparison of the SCFA levels (mM), compared using the Kruskal-Wallis test followed by Dunn’s analysis (* comparison with all the groups; # comparison with HFHC+AKK group). *#*p<*0.01.
